# Effects of Alcohol Withdrawal on Sleep Macroarchitecture and Microarchitecture in Female and Male Rats

**DOI:** 10.3389/fnins.2022.838486

**Published:** 2022-06-10

**Authors:** Marissa R. Jones, Adam J. Brandner, Leandro F. Vendruscolo, Janaina C. M. Vendruscolo, George F. Koob, Brooke E. Schmeichel

**Affiliations:** ^1^Department of Biomedical Sciences, Quillen College of Medicine, East Tennessee State University, Johnson City, TN, United States; ^2^Neurobiology of Addiction Section, Integrative Neuroscience Research Branch, National Institute on Drug Abuse, Intramural Research Program, Baltimore, MD, United States

**Keywords:** ethanol, alcohol use disorder, alcohol dependence, rapid eye movement, sleep spindle, macroarchitecture, microarchitecture

## Abstract

The prevalence of sleep disruptions is higher among people with alcohol use disorder (AUD), particularly during alcohol withdrawal, compared to non-AUD individuals. Although women generally have a higher risk of developing sleep disorders, few studies have investigated sex differences in sleep disruptions following chronic alcohol exposure. The present study examined sleep macroarchitecture (time spent asleep or awake and sleep onset latency) and microarchitecture (bout rate and duration and sleep spindle characterization) prior to alcohol vapor exposure (baseline), during acute withdrawal, and through protracted abstinence in female and male rats. Females and males showed reduced time in rapid eye movement (REM) sleep during acute withdrawal, which returned to baseline levels during protracted abstinence. REM sleep onset latency was decreased during protracted abstinence in females only. Furthermore, there was a sex difference observed in overall REM sleep bout rate. Although there were no changes in non-REM sleep time, or to non-REM sleep bout rate or duration, there was an increase in non-REM sleep intra-spindle frequency during acute withdrawal in both females and males. Finally, there was increased wakefulness time and bout duration during acute withdrawal in both females and males. The results demonstrate both macroarchitectural and microarchitectural changes in sleep following chronic alcohol exposure, particularly during acute withdrawal, suggesting the need for therapeutic interventions for sleep disturbances during withdrawal in individuals with AUD. Furthermore, sex differences were observed in REM sleep, highlighting the importance of including both sexes in future alcohol-related sleep studies.

## Introduction

Alcohol, a central nervous system depressant, can have varying effects on sleep quality. During acute intoxication, alcohol initially acts as a sedative-hypnotic, resulting in shortened sleep onset latency, increased slow-wave sleep (SWS; or non-rapid eye movement; NREM), decreased sigma power (typically associated with sleep spindles in NREM sleep), and dose-dependent suppression of rapid eye movement (REM) sleep during the first half of the night that can rebound once blood alcohol levels (BALs) have decreased during the second half of the night (Rundell et al., 1972; MacLean and Cairns, 1982; Dijk et al., 1992; Feige et al., 2006; Sagawa et al., 2011; Chan et al., 2015). During the second half of the night, wakefulness and sleep stage transitions are increased, resulting in disrupted sleep.

Alcohol use disorder (AUD) is a chronic disease characterized by the inability to stop or control alcohol consumption despite the social and health consequences to the individual. Individuals with AUD may experience cravings and physical and/or psychological withdrawal symptoms when the acute effects of alcohol subside, which may include anxiety, depression, trouble sleeping and restlessness among many others (Reus et al., 2018). Sleep disturbances observed with chronic alcohol use include extended sleep onset latency, decreased quality of restful sleep and increased fragmented REM sleep (Mukherjee and Simasko, 2009; Thakkar et al., 2010, 2015). A growing number of investigations into the association of AUD and sleep disturbances reveal an important, but complex, clinical comorbidity. The prevalence of sleep disturbances is higher among people with AUD compared to non-AUD individuals. Rates of insomnia, a common sleep disorder indicated as difficulty falling or staying asleep, is greater than 90% among those individuals with AUD (Brower, 2001; Brower and Perron, 2010). Persistent sleep disturbances, altered sleep architecture, and insomnia are clinically noted co-occurrences in individuals with AUD across the stages of the addiction cycle, including binge/intoxication, withdrawal/negative affect, and preoccupation/anticipation (Koob and Colrain, 2020). Individuals will experience an increase in SWS following alcohol binge/intoxication and a subsequent decrease in SWS during the withdrawal/negative affect state of addiction, while there is no definitive trend with REM sleep. During the preoccupation/anticipation stage, there seems to be a trend of decreased SWS that starts to partially recover during alcohol abstinence, while REM is either increased or decreased in patients with AUD compared to controls (for review, Koob and Colrain, 2020). Generally, individuals with AUD show relatively limited recovery of sleep disturbances in the first month of abstinence, and persistent sleep dysfunction has been reported despite several months of alcohol abstinence. Notably, insomnia has been shown to increase the likelihood of relapse in some long-term alcohol-abstinent individuals with AUD (Brower, 2015), indicating a possible bidirectional relationship between sleep dysfunction and AUD. Thus, there is increasing interest in investigating sleep abnormalities in individuals with AUD from the perspective of treatment success.

Cortical hyperarousal associated with insomnia has been found to be related to alterations in sleep protection mechanisms, such as sleep spindle activity during NREM sleep (Normand et al., 2016). Periods of NREM sleep contain low-frequency delta (0.5–3 Hz) waves with distinct, rapid bursts of high-frequency waves in the sigma band (10–15 Hz), referred to as sleep spindles. Sleep spindle microarchitecture is associated with general forms of intelligence and memory functioning (Bódizs et al., 2005) and reflects NREM sleep protective mechanisms (Kim et al., 2012), such as sensory gating (for review, see Fernandez and Lüthi, 2020). It remains to be determined whether sleep disturbances in individuals with AUD are also related to changes in sleep spindle characteristics.

Sex differences of alcohol effects on sleep are rarely reported. In humans, women tend to experience more subjective sedation at a lower BAL than men (Jünger et al., 2016), and more objective sleep disturbances at the same BAL than men (Arnedt et al., 2011). In contrast to this, another study observed no sex difference in sleep architecture following acute alcohol consumption (Chan et al., 2013). Notably, females also show a higher risk for non-alcohol-related sleep disorders, including insomnia, compared to males (Zhang and Wing, 2006). Most preclinical alcohol-related sleep studies are limited to male rodents, thus neglecting sex as a variable. However, one study demonstrated not only overall sleep deficits in alcohol-dependent mice, but also a sex difference in sleep regulation, specifically showing increased sleep onset latency in female mice and disrupted sleep maintenance in male mice during acute withdrawal (Huitron-Resendiz et al., 2018).

Preclinical models investigating the relationship of alcohol and sleep disruptions also largely focus on effects of acute, rather than chronic, alcohol on sleep architecture. Studies using chronic alcohol administration in male rodents have reported inconsistencies in effects on REM sleep, with evidence of increased REM sleep (Veatch, 2006), fragmentation of REM sleep (Mukherjee and Simasko, 2009; Thakkar et al., 2010, 2015) and evidence of no impact on REM sleep (Sharma et al., 2021). Results also vary for SWS, showing suppressed SWS following chronic alcohol exposure in some cases (Ehlers and Slawecki, 2000; Huitron-Resendiz et al., 2018; Sharma et al., 2021) and increased SWS in others (Mukherjee and Simasko, 2009; Sanchez-Alavez et al., 2019), depending on the time of data collection, as well as chronicity, length, and routes of alcohol exposure.

Although discrepancies between study designs make direct comparisons difficult, sex differences in sleep disturbances are present and measurable in rodent models of alcohol intake. It appears that the degree to which sleep architecture is altered in rodents is highly dependent on four key experimental design elements: length/route of alcohol exposure, temporal dynamics of alcohol withdrawal/dependence, data collection time of day, and sex of the animal. The present preclinical study sought to characterize sleep dysfunction during acute alcohol withdrawal and into protracted abstinence in both female and male rats following chronic ethanol vapor exposure, a reliable model of alcohol dependence, showing both predictive and construct validity, that produces somatic and motivational signs of withdrawal resembling those in humans with AUD (Heilig et al., 2010; Vendruscolo and Roberts, 2014; Vendruscolo et al., 2015; Aoun et al., 2018). We predicted that changes to REM and NREM sleep architecture would occur particularly during acute withdrawal from chronic alcohol exposure and would be more pronounced in females. The current study employed not only more traditional sleep macroarchitecture measures (i.e., time spent asleep and sleep onset latency), but also sleep microarchitecture measures (i.e., sleep bout and NREM sleep spindles characterization), as potential indicators of alcohol-related sleep dysfunction, and to provide additional insights into the disruption of sleep that occurs during acute withdrawal and protracted abstinence.

## Materials and Methods

### Subjects

Eight adult male and twelve adult female Wistar rats were obtained from Charles River Laboratories (Raleigh, NC, United States) and housed in pairs under a reversed light/dark (12:12 h) cycle with *ad libitum* access to food and water within a temperature-controlled vivarium (22–23°C). At the start of the experiment, male and female rats weighed approximately 250–350 g, with approximate age-matching between sexes (9–10 weeks old). Their body weights were monitored throughout the course of the experiment (Supplementary Figure 1A). All procedures conducted during this experiment were approved by the Animal Care and Use Committee of the National Institute on Drug Abuse Intramural Research Program.

### Surgery

Animals were anesthetized using isoflurane and implanted with F40-EET telemetry devices (Data Sciences International; DSI; St. Paul, MN, United States) in the lower right abdomen and secured into the muscle wall. Two electromyography (EMG) and electroencephalography (EEG) leads each were secured into the nuchal muscle and skull (A/P: +1.0 mm, M/L: +1.0 mm; A/P: −2.0 mm, M/L: −1.0 mm), respectively. During the 3-day post-operative period, animals were treated with meloxicam (1 mg/kg) and gentamicin (1 mg/kg). Animals recovered for 4–6 weeks before the experimental procedures continued.

### Chronic, Intermittent Ethanol Vapor

Following recovery from surgery and baseline recordings, all animals were pair-housed in home cages that were placed inside the chronic ethanol vapor chambers to induce alcohol dependence as described previously (Gilpin et al., 2008; Vendruscolo and Roberts, 2014). The chronic, intermittent ethanol vapor (CIEV) model of alcohol dependence elicits rapid tolerance, without detrimental health effects, typically within 2–4 weeks. Six to eight hours after the end of exposure to CIEV, rats exhibit both somatic and motivational signs of withdrawal, and the motivational signs can still be observed 3–5 weeks into alcohol withdrawal (Gilpin et al., 2009; Koob et al., 2014; Vendruscolo and Roberts, 2014). Mean body weights at the start of CIEV exposure were 277.4 ± 6.6 and 460.9 ± 6.2 g for females and males, respectively (Supplementary Figure 1A; baseline). Here, rats underwent cycles of CIEV for 12 h ethanol vapor OFF [zeitgeber time (ZT) 0–12, where ZT 0 indicates beginning of light phase] and 12 h ethanol vapor ON (ZT 13–24, where ZT 13 indicates beginning of dark phase). The amount of alcohol vaporized was independently controlled per sex to maintain the desired BAL in the range of 150–225 mg/dL, regardless of body weight. We targeted BALs in this range as it is known to cause both somatic (e.g., rigidity, irritability, hyperalgesia) and motivational (e.g., anxiety-like behavior, increased alcohol self-administration) signs of withdrawal (Gilpin et al., 2008, 2009; Vendruscolo and Roberts, 2014; Glover et al., 2021). Animals were tested weekly for BALs to determine intoxication induced by CIEV exposure. Mean BALs of 195.2 ± 13.4.9 and 226.0 ± 17 mg/dL were achieved for the females and males, respectively, after 4 weeks of CIEV exposure (Supplementary Figure 1B; acute withdrawal). Mean body weight after 4 weeks of CIEV exposure were 303.2 ± 6.0 and 550.1 ± 15.9 g for females and males, respectively (Supplementary Figure 1A).

### Electrophysiological Recording and Analysis

**FIGURE 1:**
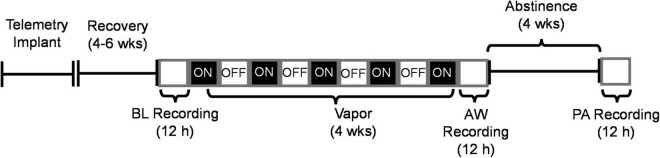
Experiment timeline depicting recordings of sleep during baseline (BL), acute withdrawal from alcohol (AW) and following 4 weeks of protracted abstinence (PA). The open white boxes represent the light phase (ZT 0–12 h) and the black boxes represent the dark phase (ZT 12–24 h). CEIV occurred during the dark phase (as indicated by ON), and ethanol was turned OFF during the light phase. All recordings were conducted during the light phase from ZT 0–12 h.

**FIGURE 2:**
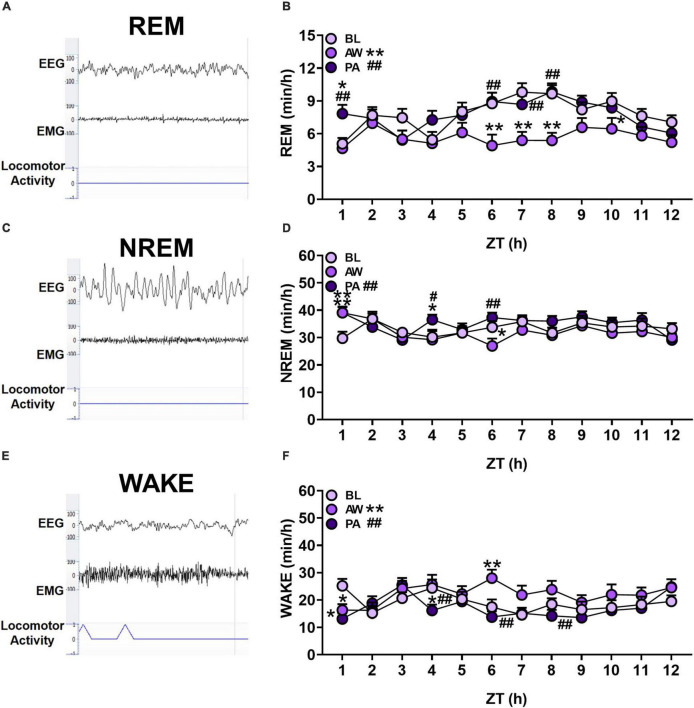
**(A,C,E)** Representative EEG and EMG traces (μV), and locomotor activity counts of each sleep/wakefulness state (REM, NREM and WAKE). **(B,D,F)** The effects of alcohol withdrawal state (baseline [BL]: light purple circles; acute withdrawal [AW]: medium purple circles; and protracted abstinence [PA]: dark purple circles) on mean total time (minutes; + SEM) for each hour spent in each sleep/wakefulness state (REM, NREM and WAKE) for females and males combined. *p < 0.05, **p < 0.01 vs. BL; ^#^*p* < 0.05, ^##^*p* < 0.01 vs. AW.

All sleep/wakefulness recordings were collected from ZT 0–12. Rats were first habituated to their home cages within the ethanol vapor chambers (vapor OFF), and baseline data were collected before any exposure to alcohol. Following baseline, rats were exposed to CIEV for 4 weeks. For acute alcohol withdrawal, recordings started at ZT 0 immediately following the last vapor exposure (i.e., the preceding ZT 13–24). BALs are expected to decrease continually from the targeted 150–225 mg/dL at time ZT 0 and reach negligible BALs in about 4.5 h as shown previously in dependent male rats (Gilpin et al., 2009). Protracted abstinence data were collected 4 weeks following acute withdrawal (see Figure 1 for experiment timeline). Sleep macroarchitecture (i.e., time spent in each sleep/wakefulness state and sleep onset latency) and microarchitecture (bout rate, bout duration, and sleep spindle characteristics) were analyzed for each timepoint (baseline, acute withdrawal, and protracted abstinence). For telemetry recordings, the EEG and EMG signals were amplified using a Data Exchange Matrix and acquired using Ponemah software (DSI, version 5.2). Each 12-h recording was digitized at a rate of 500 Hz and then decomposed using a Fast Fourier Transformation to parse out each wavelength into 0.25 Hz bins. A bandpass filter (0.5–50 Hz) was used to exclude any incoming noise at high and low frequencies. EEG/EMG signals were manually scored in 2-s epochs into three general vigilance/sleep states: rapid eye-movement (REM) sleep, non-rapid eye-movement (NREM) sleep, or wakefulness (see Figures 2A,C,E for representative traces) using Neuroscore software (DSI, version 3.3). Transitioning into a different sleep state occurred only if the new state lasted 10 s or more. If the new sleep/wakefulness state was shorter than 10 s, then the animal did not undergo a transition, and the most recent scored state persisted. The time the rat spent (minutes) in a specific sleep/wakefulness state was used to calculate sleep onset latency (minutes from ZT 0 h to maintain REM or NREM sleep for at least 1 min), average bout rate (number of bouts/minute spent in sleep state) and average bout duration (number of minutes/bout). Sleep spindles occurring in NREM sleep were detected and analyzed using the Detect Spindles EEGLAB plugin (Ray et al., 2015), which is a validated, automated method of detecting sleep spindles. Complex Demodulation was used to extract power (in μV) within the sigma frequency band (11–16 Hz), and the signal was transformed into z-scores and utilized a 60-s sliding window to account for intra- and inter-individual differences in spindle characteristics. Parameters for spindle detection consisted of a central frequency of 13.5 Hz, a bandwidth of 5 Hz, a minimum of 0.25 s between each spindle event, and a minimum duration of 0.49 s for each spindle event. Spindle duration, frequency, and peak amplitude (μV) were extracted across all NREM sleep epochs over the 12-h recording. The average duration and intra-spindle frequency of sleep spindles for each hour were calculated in MATLAB. Additional number (counts) and amplitude measures for sleep spindles can be found in Supplementary Figure 5. Spectral power was examined for REM and NREM sleep over the 12-h recording using the MATLAB plugin EEGLAB (Delorme and Makeig, 2004; see Supplementary Figure 6).

### Statistical Analysis

All data are expressed as mean and standard error of the mean (+SEM). Statistical analyses were performed using GraphPad Prism software (version 8.02). The time spent in each sleep/wakefulness state (REM, NREM, and wakefulness) for each hour within the 12-h period (ZT 1–12) was collapsed across sex (no sex differences were observed in an analysis of the whole 12-h period; see Supplementary Section 1.2). The time spent in each sleep/wakefulness state within each hour was then analyzed using a repeated-measures two-way analysis of variance (ANOVA) with alcohol withdrawal state (baseline, acute withdrawal, and protracted abstinence) as the within-subjects factor and time as a between-subjects factor. Bout rates, bout duration, and sleep onset latency for REM and NREM sleep states over the whole 12-h period (ZT 0–12) were each analyzed using a repeated-measures two–way ANOVA, with alcohol withdrawal state (baseline, acute withdrawal, and protracted abstinence) as the within-subjects factor and sex as a between-subjects factor. Due to missing data points where values equaled 0 (i.e., the rat had no sleep spindles during that hour), spindle duration and frequency were analyzed using a mixed-effects model, applying a restricted maximum likelihood approach to analyze repeated-measures. When appropriate, *post hoc* comparisons were performed using Tukey’s multiple-comparison test. For all analyses, α < 0.05 was considered statistically significant.

## Results

### Effects of Acute Alcohol Withdrawal on Sleep Macroarchitecture

#### Time Spent in Rapid Eye Movement and Non-rapid Eye Movement Sleep

The time spent in each sleep/wakefulness state for each hour of the light cycle (ZT 1–12 h) for females (*n* = 12) and males (*n* = 8) was determined during baseline, acute withdrawal, and protracted abstinence (for overall total and percent time, see Supplementary Figure 2). For the time spent in REM sleep, there was a significant main effect of alcohol withdrawal state and time, and an interaction of withdrawal state and time [Figure 2B; Withdrawal state: *F*_(2, 38)_ = 36.16, *p* < 0.01; Time: *F*_(11, 209)_ = 3.70, *p* < 0.01; Withdrawal state × time: *F*_(22, 418)_ = 1.77, *p* < 0.05]. *Post hoc* analyses revealed a significant decrease in the time spent in REM sleep during acute withdrawal compared to baseline (*p* < 0.01), which returned to baseline levels during protracted abstinence (*p* < 0.01). Specifically, *post hoc* analyses revealed a significant decrease in REM sleep during acute withdrawal compared to baseline during ZT 6–8 h (*p* < 0.01) and 10 h (*p* < 0.05). There was a subsequent increase during protracted abstinence compared to baseline (*p* < 0.05) and acute withdrawal (*p* < 0.01) at ZT 1 h, and compared to acute withdrawal at ZT 6, 7, and 8 h (*p* < 0.01). These data demonstrate REM sleep alterations during acute withdrawal that appears to return to baseline within 4 weeks of protracted abstinence.

In the examination of the time spent in NREM sleep for each hour within the 12-h period, there was a significant main effect of alcohol withdrawal state and time, and an interaction of withdrawal state and time [Figure 2D; Withdrawal state: *F*_(2, 38)_ = 6.60, *p* < 0.01; Time: *F*_(11, 209)_ = 2.91, *p* < 0.01; Withdrawal state × time: *F*_(22, 418)_ = 2.02, *p* < 0.01]. *Post hoc* analyses revealed a significant increase in the time spent in NREM sleep during protracted abstinence compared to acute withdrawal (*p* < 0.01). There was a significant increase during acute withdrawal compared to baseline during ZT 1 h (*p* < 0.01), which subsequently decreased in ZT 6 h (*p* < 0.05). During protracted abstinence, there was a significant increase in the time spent in NREM sleep compared to baseline at ZT 1 h (*p* < 0.01), with a significant increase in NREM sleep during protracted abstinence compared to baseline and acute withdrawal (*p* < 0.05) in ZT 4 h and compared to acute withdrawal in ZT 6 h (*p* < 0.01). These data suggest that there may be compensatory NREM sleep during protracted abstinence.

The analysis of the time spent in wakefulness in each hour indicated a significant main effect of alcohol withdrawal state and time, and an interaction of alcohol withdrawal state and time for wakefulness state [Figure 2F; Withdrawal state: *F*_(2, 38)_ = 15.58, *p* < 0.01; Time: *F*_(11, 209)_ = 2.83, *p* < 0.01; Withdrawal state × time: *F*_(22, 418)_ = 2.30, *p* < 0.01]. *Post hoc* analyses revealed a significant increase in the overall time spent in wakefulness during acute withdrawal compared to baseline (*p* < 0.01), with a decrease in the overall time spent in wakefulness during protracted abstinence compared to acute withdrawal (*p* < 0.01). Additionally, there was a significant decrease in ZT 1 h only (*p* < 0.05), which increased in ZT 6 h (*p* < 0.01). During protracted abstinence, there was a decrease compared to baseline in ZT 1 h (*p* < 0.01), and compared to acute withdrawal in ZT 4, 6, and 8 h (*p* < 0.01). These data reveal an increased time spent in wakefulness, potentially at the expense of REM sleep, during acute withdrawal.

#### Sleep Onset Latency

REM and NREM sleep onset latency was determined from the beginning of the light phase (i.e., ZT 0) as the time the animal transitioned into the sleep state and maintained that sleep state for at least 1 min during baseline, acute withdrawal, and protracted abstinence (see Table 1). For the examination of REM sleep onset latency, one subject was excluded from the analysis as an outlier (i.e., > 3 standard deviations from the mean). There was a significant effect of alcohol withdrawal state and an interaction of withdrawal state and sex for REM sleep onset latency [Withdrawal state: *F*_(2, 34)_ = 5.26, *p* **<** 0.05; Sex: *F*_(1, 17)_ = 0.0002, *p* = 0.99; Withdrawal state × sex: *F*_(2, 34)_ = 3.74, *p* < 0.05]. *Post hoc* analyses revealed that females had a shorter onset to REM sleep during protracted abstinence compared to baseline (*p* < 0.01). In contrast, there was no effect of withdrawal state or sex on NREM sleep onset latency [Withdrawal state: *F*_(2, 36)_ = 1.47, *p* = 0.24; Sex: *F*_(1, 18)_ = 3.44, *p* = 0.08; Withdrawal state × Sex: *F*_(2, 36)_ = 0.17, *p* = 0.84]. These data indicate a significant decrease in REM sleep onset latency in females at least 4 weeks following the last CIEV exposure.

**TABLE 1:** REM and NREM sleep onset latency.

Sleep state	Sex	Withdrawal state	Mean time (min)	Sig.
REM	Females	BL	43.82 (+ 3.8)	-
		AW	29.12 (+ 7.8)	-
		PA	15.85 (+ 5.3)	↓**
	Males	BL	27.8 (+ 4.1)	-
		AW	36.23 (+ 5.41)	-
		PA	26.6 (+ 3.4)	-
NREM	Females	BL	19.72 (+ 4.0)	-
		AW	10.55 (+ 3.1)	-
		PA	13.88 (+ 4.7)	-
	Males	BL	12.7 (+ 3.1)	-
		AW	7.33 (+ 1.4)	-
		PA	11.52 (+ 3.1)	-

Arrow indicates a decrease in sleep onset latency (SOL), whereas “-” indicates no change. Asterisks represent a significant main effect of alcohol withdrawal state on SOL. **p < 0.01 (two-way ANOVA, post hoc Tukey’s test). Sig., significance.

### Effects of Alcohol Withdrawal on Sleep Microarchitecture

#### Sleep/Wakefulness Bout Rate and Bout Duration

**FIGURE 3:**
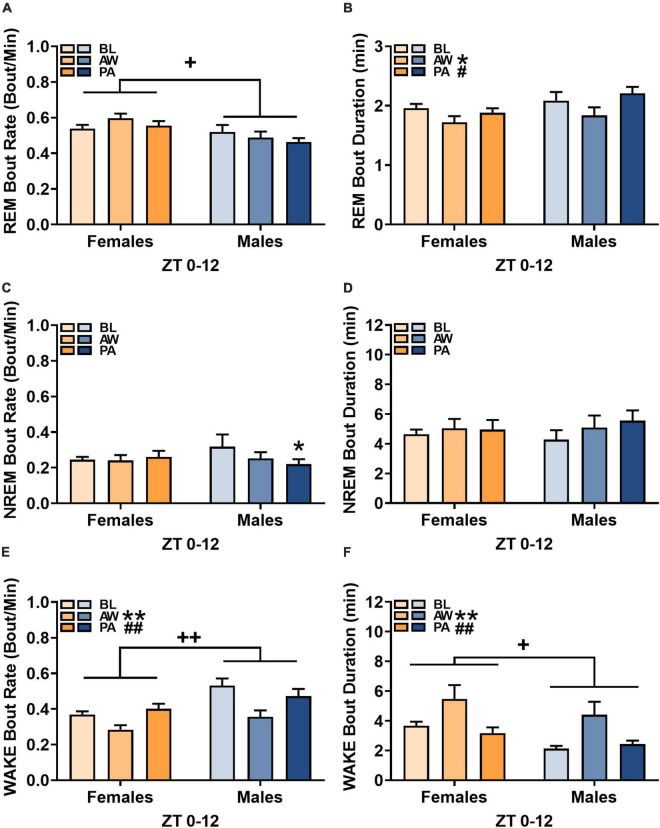
Effects of alcohol withdrawal state [baseline (BL), acute withdrawal (AW), and protracted abstinence (PA)] on **(A,C,E)** mean bout rate (bouts/minutes; + SEM) and **(B,D,F)** mean bout duration (minutes; + SEM) for each sleep/wakefulness state (REM, NREM, and WAKE) in females (orange bars) and males (blue bars) over the whole 12-h period (ZT 0–12). *p < 0.05, **p < 0.01 vs. BL; ^#^*p* < 0.05, ^##^*p* < 0.01 vs. AW; ^+^*p* < 0.05, ^++^*p* < 0.01 females vs. males.

The bout rate and bout duration for each sleep/wakefulness state over the course of the light cycle (ZT 0–12 h) for females (*n* = 12) and males (*n* = 8) was determined during baseline, acute withdrawal, and protracted abstinence (for bout duration at each hour within the 12-h period, see Supplementary Figure 3). For REM sleep bout rate, there was an effect of sex, but no effect of alcohol withdrawal state [Figure 3A; REM, Withdrawal state: *F*_(2, 36)_ = 1.11, *p* = 0.34; Sex: *F*_(1, 18)_ = 6.02, *p* < 0.05; Withdrawal state × sex: *F*_(2, 36)_ = 2.24, *p* = 0.12], indicating that females had a higher REM sleep bout rate than males. In the examination of REM sleep bout duration, there was a significant effect of alcohol withdrawal state [Figure 3B; REM sleep, Withdrawal state: *F*_(2, 36)_ = 5.53, *p* < 0.01; Sex: *F*_(1, 18)_ = 3.02, *p* = 0.10; Withdrawal state × Sex: *F*_(2, 36)_ = 0.91, *p* = 0.41]. *Post hoc* analyses revealed effects of alcohol withdrawal state on REM sleep bout duration as a significant decrease in REM sleep bout duration during acute withdrawal compared to baseline (*p* < 0.05), with a subsequent increase during protracted abstinence compared to acute withdrawal (*p* < 0.05).

For NREM sleep bout rate, there was a significant interaction of alcohol withdrawal state and sex [Figure 3C; NREM sleep, Withdrawal state: *F*_(2, 36)_ = 2.15, *p* = 0.13; Sex: *F*_(1, 18)_ = 0.11, *p* = 0.74; Withdrawal state × sex: *F*_(2, 36)_ = 3.49, *p* < 0.05]. *Post hoc* analyses revealed effects of alcohol withdrawal state on NREM sleep bout rate as a significant decrease during protracted abstinence compared to baseline (*p* < 0.01) in males only. The examination of NREM bout duration revealed no effect of alcohol withdrawal [Figure 3D; NREM, Withdrawal state: *F*_(2, 36)_ = 3.02, *p* = 0.06; Sex: *F*_(1, 18)_ = 0.01, *p* = 0.91; Withdrawal state × Sex: *F*_(2, 36)_ = 1.02, *p* = 0.37].

The examination of wakefulness bout rate revealed a significant effect of alcohol withdrawal state and a significant effect of sex [Figure 3E; Wakefulness, Withdrawal state: *F*_(2, 36)_ = 17.32, *p* < 0.01; Sex: *F*_(1, 18)_ = 9.31, *p* < 0.01; Withdrawal state × sex: *F*_(2, 36)_ = 2.31, *p* = 0.11]. *Post hoc* analyses revealed a significant decrease in wakefulness bout rate during acute withdrawal compared to baseline (*p* < 0.01) and a subsequent increase in wakefulness bout rate during protracted abstinence compared to acute withdrawal (*p* < 0.01), with females having an overall lower wakefulness bout rate than males. For wakefulness bout duration, there was a significant effect of alcohol withdrawal state and a significant effect of sex [Figure 3F; Wakefulness, Withdrawal state: *F*_(2, 36)_ = 8.05, *p* < 0.01; Sex: *F*_(1, 18)_ = 4.98, *p* < 0.05; Withdrawal state × Sex: *F*_(2, 36)_ = 0.22, *p* = 0.80]. *Post hoc* analyses revealed effects of alcohol withdrawal state on wakefulness bout duration as a significant increase in bout duration during acute withdrawal compared to baseline (*p* < 0.01), with a subsequent decrease during protracted abstinence (*p* < 0.01).

#### Non-rapid Eye Movement Sleep Spindles

**FIGURE 4:**
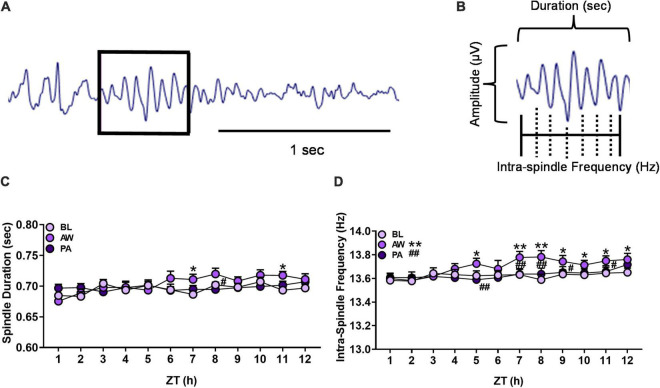
**(A)** Representative EEG trace from NREM sleep containing an identified sleep spindle (boxed) using automated spindle detection. **(B)** Diagram of sleep spindle that is characterized as a waxing and waning sine wave: amplitude (μV), sleep spindle duration (seconds), and intra-spindle frequency (Hz) oscillation cycles. **(C,D)** Effects of alcohol withdrawal state [baseline (BL): light purple circles; acute withdrawal (AW): medium purple circles; and protracted abstinence (PA): dark purple circles] on **(C)** mean spindle duration (seconds; + SEM) and **(D)** mean intra-spindle frequency (Hz; + SEM) for each hour during NREM sleep for females and males combined. *p < 0.05, **p < 0.01 vs. BL; ^#^*p* < 0.05, ^##^*p* < 0.01 vs. AW.

The duration and intra-spindle frequency of sleep spindles (see Figures 4A,B) during NREM sleep for each hour of the light cycle (ZT 1–12 h) for females (*n* = 12) and males (*n* = 8) were determined during baseline, acute withdrawal, and protracted abstinence (for the number and amplitude of sleep spindles, see Supplementary Figure 5). Spindle duration was further analyzed for each hour within the 12-h period. There was a significant interaction of alcohol withdrawal state by time [Figure 4C; Withdrawal state: *F*_(2, 38)_ = 2.03, *p* = 0.14; Time: *F*_(11, 209)_ = 3.62, *p* < 0.01; Withdrawal state × time: *F*_(22, 416)_ = 1.93, *p* < 0.01]. *Post hoc* analyses revealed effects of alcohol withdrawal state as a significant increase in spindle duration during acute withdrawal compared to baseline (*p* < 0.01) in ZT 7 and 11 h (*p* < 0.05), with a significant decrease in spindle duration during protracted abstinence compared to acute withdrawal in ZT 8 h (*p* < 0.05).

The examination of spindle frequency for each hour within the 12-h period revealed a significant effect of alcohol withdrawal state and an interaction of alcohol withdrawal state [Figure 4D; Withdrawal state: *F*_(2, 38)_ = 13.34, *p* < 0.01; Time: *F*_(11, 209)_ = 6.50, *p* < 0.01; Withdrawal state × time: *F*_(22, 416)_ = 2.24, *p* < 0.01]. *Post hoc* analyses revealed effects of alcohol withdrawal state as a significant increase in spindle frequency during acute withdrawal compared to baseline in ZT 5 h (*p* < 0.05), 7 and 8 h (*p* < 0.01), and 9–12 h (*p* < 0.05). Spindle frequency decreased during protracted abstinence compared to acute withdrawal in ZT 5 h, 7 and 8 h (*p* < 0.01), and 9 and 11 h (*p* < 0.05). These data indicate a significant increase in spindle frequency during acute withdrawal which appeared to return to baseline at least 4 weeks following the last CIEV exposure.

## Discussion

The prevalence of sleep disruptions, including insomnia, during abstinence from alcohol has been shown to be a robust predictor of relapse among individuals with AUD (Brower et al., 1998; Drummond et al., 1998; Foster and Peters, 1999; Brower, 2001). There have been few preclinical models that address the influence of acute alcohol withdrawal and protracted abstinence on sleep architecture in both female and male rodents. Here, we show altered sleep macroarchitecture and microarchitecture during acute withdrawal in female and male rats following CIEV exposure. Specifically, we observed changes in sleep macroarchitecture, with a decrease in the time spent in REM sleep, but not NREM sleep, during acute alcohol withdrawal in both female and male rats. Changes in sleep were closely associated with concomitant increases in the time spent in wakefulness during acute alcohol withdrawal. These changes returned to baseline levels during 4-week protracted alcohol abstinence. Females, but not males, showed a shortened onset to REM sleep following protracted abstinence. Altered microarchitecture was also observed in that females showed an overall higher REM sleep bout rate compared to males, although there was no effect of withdrawal state on either REM sleep bout rate or duration for the total 12-h period. An inverse outcome on wakefulness bout measures was also noted, with a decrease in wakefulness bout rate and an increase in wakefulness bout duration during acute withdrawal. There was no effect of withdrawal state on NREM sleep bout duration. Although there was no change in the overall time spent in NREM sleep in either females or males, the examination of sleep spindles during NREM sleep showed an increase of intra-spindle frequency during acute alcohol withdrawal in both females and males. These results suggest that withdrawal following chronic alcohol exposure has effects on both REM and NREM sleep macroarchitecture and microarchitecture, with sex differences observed in REM sleep.

### Effects of Alcohol Withdrawal on Sleep Macroarchitecture

Each stage of the addiction cycle (binge/intoxication, withdrawal/negative affect, and preoccupation/anticipation) is associated with unique neuroadaptations and to various sleep disturbances, alterations of sleep architecture, and the development of insomnia in individuals with AUD (Koob and Colrain, 2020). A primary focus on changes in sleep macroarchitecture during the binge/intoxication stage in the literature leaves a significant gap in our understanding of acute alcohol withdrawal and protracted abstinence on sleep among individuals with AUD. To date, there has been inconsistent clinical and preclinical evidence regarding the influence of alcohol abstinence on the time spent in REM or NREM sleep, a discrepancy largely relative to the number of days of abstinence. The time spent in REM sleep in recently or long-term abstinent individuals with AUD compared to controls has been reported to either not change or increase, whereas the time in NREM sleep increases or decreases, but largely has been shown to decrease (for review, see Koob and Colrain, 2020). Similar variabilities in the time spent in REM and NREM sleep have been reported in preclinical rodent studies, depending on the type and length of alcohol exposure, and the number of days of abstinence (Ehlers and Slawecki, 2000; Veatch, 2006; Thakkar et al., 2010, 2015; Sanchez-Alavez et al., 2019; Sharma et al., 2021). The current studies used a 4-week exposure to ethanol vapor during the dark cycle, with sleep recorded during the light cycle prior to and during acute withdrawal, and also following protracted abstinence. Somatic and motivational signs of withdrawal in rats have been observed in as few as 2 h after CIEV exposure (for review, see Vendruscolo and Roberts, 2014), with brain alcohol levels returning to baseline by 4.5 h post-CIEV (Gilpin et al., 2009). The current studies found significant changes in sleep macroarchitecture largely in the second half of the sleep/light cycle while the rats were in acute withdrawal.

The time spent in REM sleep decreased in both females and males during acute alcohol withdrawal with the changes occurring during the ZT 6, 7, 8, and 10 h time points. In contrast, decreased REM sleep has been observed in humans in the first half of the sleep cycle following acute alcohol intoxication, likely due to initially higher BALs and increased time in SWS (Rundell et al., 1972; Prinz et al., 1980). The decrease in REM sleep during acute withdrawal in the current studies returned to baseline levels following protracted abstinence. These findings are consistent with a previous study showing a decrease in REM sleep in male rats 1 day into withdrawal following chronic alcohol exposure, followed by a REM sleep rebound by day 3 of alcohol withdrawal (Mendelson et al., 1978). This contrasts with a study conducted in male mice chronically exposed to ethanol vapor, showing no change in the percent time spent in REM sleep 1 day into alcohol withdrawal, and a subsequent increase in the percentage of REM sleep by day 3 of alcohol withdrawal (Veatch, 2006). It is notable that both of these prior studies used a significantly shorter, albeit repeated, exposure to alcohol than the current studies, making direct comparisons difficult. The current studies also showed a return of REM sleep to baseline levels during protracted abstinence compared to acute withdrawal in both females and males. The decrease in REM sleep during acute withdrawal may be a product of repeated stress associated with alcohol withdrawal experienced during CIEV. Likewise, other studies have shown that repeated restraint or footshock stress can cause a decrease in both NREM and REM sleep in female and male rats (Jean Kant et al., 1995; Papale et al., 2005; Hegde et al., 2011; Gargiulo et al., 2021). Furthermore, it has been hypothesized that REM sleep is associated with the processing and stabilization of memory transformed into long-term memory during SWS (for review, Rasch and Born, 2013). Thus, whereas an increase in REM sleep may facilitate the consolidation of aversive alcohol withdrawal memory acquired during wakefulness, it is possible that the inverse, such as the reduction in REM sleep during acute withdrawal observed herein, may be protective to some degree. It is also possible that rats experienced rebound sleep during the dark phase (not recorded here), which would mimic the REM sleep rebound observed in humans that is used as a predictor for the propensity for relapse (Gillin et al., 1994; Brower et al., 1998; Clark et al., 1998). Indeed, a suppression of REM sleep during the light phase and a subsequent increase in REM sleep during the dark phase 3 weeks following chronic alcohol cessation in male rats have been reported (Kubota et al., 2002).

In regards to NREM sleep macroarchitecture, we found no changes in overall time spent in NREM sleep compared to baseline, although there was an increase in NREM sleep during protracted abstinence compared to acute alcohol withdrawal. There are inconsistent reports in the literature of preclinical chronic alcohol models addressing the impact of acute alcohol withdrawal or protracted abstinence on NREM sleep and SWS, ranging from a decrease in NREM sleep during acute withdrawal (Veatch, 2006; Sharma et al., 2021) to having no impact on the time spent in SWS in either acute withdrawal or protracted abstinence (Ehlers and Slawecki, 2000) in males. The differences in the route of chronic alcohol administration and the time of withdrawal between each of these studies may be contributing to opposing findings. Additionally, considering that rats spend 50–70% of their time asleep during the light cycle, subtle changes in the total time spent in NREM sleep may be difficult to capture, suggesting that other measures of NREM sleep microarchitecture may also be useful.

The current studies found a sex-specific effect on REM sleep onset latency, as demonstrated by shortened REM sleep onset latency following protracted abstinence in females. There were no effects of alcohol withdrawal on NREM sleep onset latency in either females or males. This contrasts with previous reports of an increase in NREM sleep onset latency during acute withdrawal (Sharma et al., 2021) or protracted abstinence (Ehlers and Slawecki, 2000) in males, or no change in REM or NREM sleep onset latency during early abstinence in females (Amodeo et al., 2020). Typically, a shortened sleep onset latency indicates increased tiredness and sleep pressure, potentially due to a prior sleep deficit or some underlying chronic sleep disorder. Clinically, it has been reported that shortened REM sleep onset latency is associated with higher relapse rates in individuals with AUD (Gillin et al., 1994; Brower et al., 1998). Investigating whether a shortened REM sleep onset latency during protracted abstinence is associated with the reinstatement of alcohol seeking or another rodent alcohol-relapse model is an important topic for future studies.

The current studies demonstrate an effect of alcohol withdrawal after chronic alcohol exposure on sleep macroarchitecture in both female and male rats, as shown by a reduced time spent in REM sleep in females and males during acute withdrawal, and decreased REM sleep onset latency during protracted abstinence in females. These results highlight the importance of REM sleep and including both sexes into future studies investigating alcohol withdrawal effects on sleep.

### Effects of Alcohol Withdrawal on Sleep Microarchitecture

Although standard sleep measures (i.e., macroarchitecture), such as the time spent asleep or awake and sleep onset latency, are important, they do not completely characterize sleep dysfunction. Thus, the current studies considered further analyses of sleep microarchitectural components, including sleep bout and spindle characterization, during acute alcohol withdrawal and following protracted abstinence from alcohol. The current studies revealed an overall higher REM sleep bout rate in females compared to males, although there was no effect of withdrawal state on REM sleep bout measures. However, the examination of hourly data for females and males combined showed an overall decrease in REM sleep bout duration during acute withdrawal that returned to baseline levels during protracted abstinence (see Supplementary Figure 3). This decrease in REM sleep bout duration may reflect the reduced time spent in REM sleep, or could be indicative of reduced quality of REM sleep, particularly in the latter half of the light/sleep cycle. Further REM sleep bout analysis is warranted to determine whether REM sleep is asynchronous, or fragmented, during acute withdrawal. An inverse outcome on wakefulness bout measures was also noted, with a decrease in wakefulness bout rate and increase in wakefulness bout duration during acute withdrawal. Finally, although there was a significant decrease in NREM sleep bout rate during protracted abstinence in males, suggesting a longer time spent in each bout of NREM sleep, there was no significant effect of withdrawal state on NREM sleep bout duration detected in either males or females. Our findings are in contrast to another study in which they observed decreased NREM sleep bout duration during acute withdrawal following CEIV (Sanchez-Alavez et al., 2019), although this could be due to differences in length of alcohol exposure.

Although there was no significant effect of alcohol withdrawal on the overall time spent in NREM sleep, the examination of sleep spindles, a hallmark of NREM sleep, revealed changes in NREM sleep microarchitecture in both female and male rats. During acute alcohol withdrawal, despite no overall change in spindle duration, there was an increase in intra-spindle frequency (i.e., the rate of spindle oscillation) that returned to baseline levels during protracted abstinence. Sleep spindles are considered to be sleep-protective elements, shielding NREM sleep from spontaneous sensory input or disruptions, possibly at the expense of REM sleep (Fernandez and Lüthi, 2020; Sippel et al., 2020). Thus, the lack of change in the time spent in NREM sleep and the decreased time in REM sleep found in the current study may reflect the influence of NREM sleep spindles during acute withdrawal from alcohol. Stress has consistently been shown to be a profound contributing factor to AUD, particularly during alcohol withdrawal in both humans and rodents (Becker, 2012; Tunstall et al., 2017; Vendruscolo and Koob, 2020). Both clinical and preclinical studies have demonstrated a consistent interaction between physiological systems of stress/overactive arousal and sleep (Rodenbeck and Hajak, 2001; Riemann et al., 2015; Kalmbach et al., 2018; Lo Martire et al., 2020) including increased spindle frequency in individuals with post-traumatic stress disorders (Van Reeth et al., 2000; Wang et al., 2020). Therefore, the increased stress response associated with acute alcohol withdrawal may contribute to the increase in intra-spindle frequency observed in the current studies. However, the role of stress systems in sleep dysfunction associated with alcohol withdrawal has yet to be elucidated.

Together, these results indicate a role for acute alcohol withdrawal in the changes in sleep microarchitecture. Although standard sleep measures, such as the time spent asleep or awake, are important tools, restricting sleep measures to standard macroarchitecture components may be limiting our full understanding of alcohol-related sleep dysfunction. Here, we showed that microarchitectural changes not only in the continuity of sleep state, but also in sleep-spindle elements. These indices of sleep microarchitectural change during alcohol withdrawal and may be potential sleep biomarker candidates for the prediction of AUD treatment outcomes in future studies.

### Limitations

A limitation of the present study is that we did not track estrous cycle in female rats throughout the experiment. The estrous cycle has been reported to influence sleep patterns, with proestrus being associated with reduced REM sleep, enhanced waking, and increased sleep spindle density at the end of the light cycle when progesterone and estradiol are highest (Schwierin et al., 1998; Swift et al., 2019). Regarding alcohol drinking and CEIV-induced alcohol drinking, we have reported that the estrous cycle had minimal influence on these behaviors (Priddy et al., 2017). Hormonal effects during estrous cycle also seem to be most influential during acquisition of drug-taking behavior and less so once compulsive drug-taking has been established (Becker and Koob, 2016). However, whether there is an interaction effect between passive alcohol exposure and estrous cycle on sleep architecture remains to be determined.

An additional possible limitation of the current studies lies in the sleep spindle characterization. It has been reported that alcohol decreases sigma band power (10–15 Hz), typically associated with sleep spindles, during NREM sleep in humans (Dijk et al., 1992; Chan et al., 2015). In analyzing the sigma band during NREM sleep for spindles in the current studies, we did not differentiate between slow (<12 Hz) and fast (>12 Hz) oscillating spindles, which largely vary based on topographical location (Terrier and Gottesmann, 1978; Gandolfo et al., 1985; Sitnikova et al., 2014). It remains to be seen whether the distinction between slow and fast spindles has any functional significance, although evidence suggests that fast oscillating sleep spindles are associated with sleep-dependent memory processing (Mölle et al., 2011), which may have implications for processes of extinction and thus, addiction recovery (Feld and Born, 2020; Valentino and Volkow, 2020). This is an exciting future direction for sleep microarchitecture research in the field of addiction.

## Summary and Conclusion

In summary, we demonstrated alterations of sleep macroarchitecture and microarchitecture during acute alcohol withdrawal and protracted abstinence in female and male rats following chronic alcohol exposure. We observed a decrease in REM sleep during acute alcohol withdrawal in both females and males. This decrease in REM sleep during alcohol withdrawal may reflect stress associated with the experience of withdrawal, as REM sleep returned to baseline during protracted abstinence. Females had shortened REM sleep onset latency during protracted abstinence, indicating a sex difference. Additionally, we demonstrated altered sleep microarchitecture in the forms of REM sleep bout measures and NREM sleep spindle frequency, thus providing additional insights into the effects of alcohol withdrawal on sleep that are not captured using macroarchitecture measures. The observed sex differences in sleep architecture in the current studies also highlight the necessity of addressing sex differences in preclinical models to test the effectiveness of pharmacological treatments for alcohol dependence and its associated sleep dysfunction for both females and males. The incorporation of acute withdrawal and protracted abstinence into a preclinical model of AUD may aid in identifying an optimal stage and therapeutic target for reducing the risk of relapse. Finally, future studies should aim to discover underlying neuronal mechanisms of chronic disrupted sleep and how they may contribute to perpetuating AUD. Having an established role in sleep/wakefulness modulation, a complementary interest has been growing for the hypocretin/orexin (HCRT) system in the context of addiction. HCRT-receptor antagonists, such as suvorexant and lemborexant (FDA-approved for the treatment of insomnia), may be beneficial medications to improve abstinence and AUD-treatment outcomes. Preclinical and clinical models have begun to examine the efficacy of HCRT-receptor antagonists in mitigating the impact of AUD on sleep architecture (Sanchez-Alavez et al., 2019; clinicaltrials.gov, NCT04229095). Ultimately, identifying the influence of acute alcohol withdrawal and protracted abstinence on sleep macroarchitecture and microarchitecture is a crucial first step for evaluating the efficacy of such treatments for AUD.

## Data Availability Statement

The raw data supporting the conclusions of this article will be made available by the authors, without undue reservation.

## Ethics Statement

The animal study was reviewed and approved by the Animal Care and Use Committee of the National Institute on Drug Abuse Intramural Research Program.

## Author Contributions

BS, LV, and GK contributed to conception, design of the study, and interpretation of the data. MJ and AB performed the acquisition of data, statistical analysis, and equally wrote the first draft of the manuscript under the supervision of BS. JV performed surgeries and assisted with data acquisition. All authors contributed to the manuscript revision and read and approved the submitted version.

## Conflict of Interest

The authors declare that the research was conducted in the absence of any commercial or financial relationships that could be construed as a potential conflict of interest.

## Publisher’s Note

All claims expressed in this article are solely those of the authors and do not necessarily represent those of their affiliated organizations, or those of the publisher, the editors and the reviewers. Any product that may be evaluated in this article, or claim that may be made by its manufacturer, is not guaranteed or endorsed by the publisher.
