# Supplementary material for: Effects of Alcohol Withdrawal on Sleep Macroarchitecture and Microarchitecture in Female and Male Rats
Source: Front Neurosci. 2022 Jun 10;16:838486. doi: 10.3389/fnins.2022.838486 (PMC9226367; doi:10.3389/fnins.2022.838486)
Supplement: Supplementary file 1 [file Data_Sheet_1.pdf]

Supplementary Materials

1. Supplementary Results

1.1. Body weights and blood alcohol levels

The body weights for females (*n* = 12) and males (*n* = 8) were examined at 4 time points; prior to telemetry surgeries, at baseline, acute withdrawal, and protracted abstinence. Body weights were analyzed using a two-analysis of variance (ANOVA) with Šidák's multiple-comparison test. For body weights, there was a significant main effect of each time and sex, and an interaction of time and sex (Figure 1A; Time: $F_{(3,54)} = 229$, $p < 0.01$; Sex: $F_{(1,18)} = 349$, $p < 0.01$; Time x Sex: $F_{(3,54)} = 73.53$, $p < 0.01$). *Post hoc* analyses revealed a significant increase in body weight at each time point, with males having an overall higher body weight than females ($p < 0.01$).

Blood alcohol levels (BALs) for females (*n* = 12) and males (*n* = 8) were measured after four weeks of CIEV exposure and analyzed using an unpaired samples t-test. There was no significant difference in the comparison of BALs between females and males (Figure 1B: $t_{(18)} = 1.41$, $p = 0.18$). This indicates that females and males had similar BALs four weeks following CIEV exposure despite differences in body weight.

1.2. Effects of alcohol withdrawal on 12-hour total time and percentage of time spent in REM, NREM, and wakefulness

The total time spent in each sleep/wakefulness state over the course of the light cycle (ZT 0-12 h) and the percentage of time for each hour (ZT 1-12 h) for females (*n* = 12) and males (*n* = 8) was determined during baseline, acute withdrawal, and protracted abstinence (**Supplementary Figure 2***)*. Total time was analyzed using a repeated-measures two-way ANOVA with alcohol withdrawal state (baseline, acute withdrawal, and protracted abstinence) as the within-subjects factor and sex as a between-subjects factor followed by a Tukey's multiple-comparison test. For total REM sleep time, there was a significant main effect of alcohol withdrawal state, but no effect of sex for the total time spent in REM sleep (Figure 2A; Withdrawal state: $F_{(2,36)} = 35.27$, $p < 0.01$; Sex: $F_{(1,18)} = 0.02$, $p = 0.90$; Withdrawal state x sex: $F_{(2,36)} = 0.78$, $p = 0.45$). *Post hoc* analyses revealed a significant decrease in REM sleep time during acute withdrawal compared to baseline ($p < 0.01$) and a subsequent significant increase in REM sleep time during protracted abstinence compared to acute withdrawal ($p < 0.01$) in both females and males. The percentage of time spent in REM sleep for each hour was examined, and there was a significant main effect of alcohol withdrawal state and an interaction of withdrawal state and time (Figure 2B; Withdrawal state: $F_{(2,38)} = 36.74$, $p < 0.01$; Time: $F_{(11,209)} = 3.63$, $p < 0.01$; Withdrawal state x time: $F_{(22,418)} = 1.82$, $p < 0.05$). *Post hoc* analyses revealed a an overall decrease in percentage of time spent in REM sleep during acute withdrawal compared to baseline ($p < 0.01$), with a subsequent increase during protracted abstinence compared to acute withdrawal ($p < 0.01$). Specifically, there was a significant decrease in the percentage of time spent in REM sleep during acute withdrawal compared to baseline in ZT 6-8 h ($p < 0.01$) and in ZT 10 h ($p < 0.01$). There was a significant increase in the percentage of REM sleep time during protracted abstinence compared to baseline ($p < 0.05$) and acute withdrawal ($p < 0.01$) in ZT 1 h, and a significant increase compared to acute withdrawal in ZT 6-8 h ($p < 0.01$).

For NREM sleep, there was a significant main effect of alcohol withdrawal state, but no main effect of sex for total time spent in NREM was observed (Figure 2C; Withdrawal state:

$F_{(2,36)} = 6.87, p < 0.01$; Sex: $F_{(1,18)} = 0.47, p = 0.50$; Withdrawal state x sex: $F_{(2,36)} = 0.52, p = 0.60$). *Post hoc* analyses revealed a significant increase in NREM sleep during protracted abstinence compared to NREM sleep during acute withdrawal ($p < 0.01$). The percentage of time spent in NREM sleep for each hour was examined, and there was a significant main effect and interaction of alcohol withdrawal state and time (Figure 2D; NREM: Withdrawal state: $F_{(2,38)} = 6.60, p < 0.01$; Time: $F_{(11,209)} = 3.00, p < 0.01$; Withdrawal state x time: $F_{(22,418)} = 2.01, p < 0.01$). *Post hoc* analyses revealed an overall increase in the percentage of NREM sleep time during protracted abstinence compared to acute withdrawal ($p < 0.01$). Specifically, there was a significant increase during acute withdrawal compared to baseline in ZT 1 h ($p < 0.01$), which decreased in ZT 6 h ($p < 0.05$). There was a significant increase during protracted abstinence compared to baseline a ZT 1h ($p < 0.01$), with a significant increase compared to acute withdrawal in ZT 4 h and 6 h ($p < 0.01$).

For the total time spent in wakefulness, there was a significant main effect of alcohol withdrawal state, but no effect of sex (Figure 2E; WAKE, Withdrawal state: $F_{(2,36)} = 16.13, p < 0.01$; Sex: $F_{(1,18)} = 0.46, p = 0.51$; Withdrawal state x sex: $F_{(2,36)} = 0.78, p = 0.47$). *Post-hoc* analyses revealed effects of alcohol withdrawal state as a significant increase in time spent in wakefulness during acute withdrawal compared to baseline ($p < 0.01$), which decreased during protracted abstinence compared to acute withdrawal ($p < 0.01$). The examination of the percentage of time spent in wakefulness for each hour revealed a significant main effect and interaction of alcohol withdrawal state and time (Figure 2F; Withdrawal state: $F_{(2,38)} = 15.74, p < 0.01$; Time: $F_{(11,209)} = 2.83, p < 0.01$; Withdrawal state x time: $F_{(22,418)} = 2.32$, $p < 0.01$). *Post hoc* analyses revealed an overall increase in the percentage of time spent in wakefulness during acute withdrawal ($p < 0.01$), which decreased during protracted abstinence ($p < 0.01$). Specifically, there was a significant decrease in the percentage of time spent in wakefulness during acute withdrawal compared to baseline in ZT 1h ($p < 0.05$), which increased during ZT 6 h ($p < 0.01$). During protracted abstinence there was a significant decrease compared to baseline in ZT 1 h and 4 h ($p < 0.01$) and compared to acute withdrawal during ZT 4 h, 6 h, and 8 h ($p < 0.01$).

1.3. Effects of alcohol withdrawal on sleep/wakefulness bout duration for each hour

The bout duration for each sleep/wake for each hour of the light cycle (ZT 1-12 h) for females ($n = 12$) and males ($n = 8$) was determined during baseline, acute withdrawal, and protracted abstinence (**Supplementary Figure 3**). Bout duration for each hour was analyzed using a repeated measure two-way (ANOVA) with alcohol withdrawal state (baseline, acute withdrawal, and protracted abstinence) as the within-subjects factor and time as a between-subjects factor followed by a Tukey's multiple-comparison test. For REM sleep bout duration, there was a significant main effect of alcohol withdrawal state and time on REM sleep bout duration over ZT 1-12 h (Figure 3A; Withdrawal state: $F_{(2,38)} = 5.27, p < 0.01$; Time: $F_{(11,209)} = 2.51, p < 0.01$; Withdrawal state x time: $F_{(22,418)} = 1.26, p = 0.19$). *Post hoc* analyses revealed an overall decrease in REM sleep bout duration during acute withdrawal compared to baseline ($p < 0.05$), with a significant increase during protracted abstinence compared to acute withdrawal ($p < 0.05$).

For NREM sleep bout duration within each hour of the 12 h period, there was a significant interaction of alcohol withdrawal state and time (Figure 3B; Withdrawal state: $F_{(2,38)} = 2.51, p = 0.09$; Time: $F_{(11,209)} = 1.66, p = 0.08$; Withdrawal state x time: $F_{(22,418)} = 2.09, p < 0.01$). *Post hoc* analyses revealed effects of alcohol withdrawal state on NREM sleep bout duration as a

significant increase in the NREM bout duration during acute withdrawal compared to baseline ($p < 0.01$) in ZT 1 h and 2 h. The NREM sleep bout duration subsequently decreased during protracted abstinence compared to baseline ($p < 0.05$) in ZT 1 h and compared to acute withdrawal ($p < 0.01$) in ZT 2 h, which increased compared to baseline in ZT 11 h ($p < 0.05$).

In the examination of wakefulness bout duration, there was a significant main effect of alcohol withdrawal state and time (Figure 3C; Withdrawal state: $F_{(2,38)} = 8.72$, $p < 0.01$; Time: $F_{(11,209)} = 1.83$, $p = 0.05$; Withdrawal state x time: $F_{(22,418)} = 1.01$, $p = 0.45$). *Post-hoc* analyses revealed an overall increase in wakefulness bout duration during acute withdrawal compared to baseline, which significantly decreased during protracted abstinence.

1.4. Effects of alcohol withdrawal on locomotor activity

Activity counts for each sleep/wakefulness state of the course of the light cycle (ZT 0-12) for females (n = 12) and males (n = 8) were measure during baseline, acute withdrawal, and protracted abstinence (**Supplementary Figure 4***)*. Activity counts over the whole 12-hour period were analyzed using a repeated-measures two–way ANOVA, with alcohol withdrawal state (baseline, acute withdrawal, and protracted abstinence) as the within-subjects factor and sex as a between-subjects factor followed by the Tukey's multiple-comparison test. There was a significant main effect of alcohol withdrawal state and of sex on activity counts (Figure 4A; Withdrawal state: $F_{(2,36)} = 8.98$, $p < 0.01$; Sex: $F_{(1,18)} = 8.24$, $p < 0.05$; Withdrawal state x sex: $F_{(2,36)} = 1.13$, $p = 0.33$). These data suggest that females had overall higher locomotor activity over the 12-hour period than males. *Post-hoc* analyses revealed a significant increase in activity counts during acute withdrawal compared to baseline ($p < 0.01$) which significantly decreased during protracted abstinence ($p < 0.01$).

Activity counts were further analyzed for each hour within the 12-hour period (ZT 1-12) using a repeated measure two-way ANOVA with alcohol withdrawal state (baseline, acute withdrawal, and protracted abstinence) as the within-subjects factor and time as a between-subjects factor. For activity counts within each hour, there was a significant main effect and interaction effect of alcohol withdrawal and time in females and males (Figure 4B; Withdrawal state: $F_{(2,38)} = 8.02$, $p < 0.01$; Time: $F_{(11,209)} = 3.08$, $p < 0.01$; Withdrawal state x time: $F_{(22,418)} = 2.82$, $p < 0.01$). *Post-hoc* analyses revealed an overall significant increase in activity counts during acute withdrawal compared to baseline ($p < 0.05$), which significantly decreased during protracted abstinence compared to acute withdrawal ($p < 0.01$). Specifically, there was a decrease in activity counts during acute withdrawal compared to baseline ($p < 0.01$) in ZT 1 h, which increased compared to baseline during ZT 6 h- 8 h ($p < 0.05$). During protracted abstinence there was a significant decrease compared to baseline in ZT 1h ($p < 0.01$), a decrease compared to acute withdrawal in ZT 4 h ($p < 0.05$), ZT 6 h-8 h ($p < 0.05$), with an increase compared to baseline in ZT 12 h ($p < 0.05$).

1.5. Effects of alcohol withdrawal on the number and amplitude of spindles in NREM sleep

The number of spindles and amplitude of sleep spindles during NREM sleep over the course of the light cycle (ZT 0-12) for males (n=8) and females (n=12) was determined during baseline, acute withdrawal, and protracted abstinence (**Supplementary Figure 5**). Due to missing data points where values equaled 0 (i.e., the rat had no sleep spindles during that hour), the number of spindles and spindle amplitude were analyzed using a mixed-effects model, applying a restricted

maximum likelihood approach to analyze repeated measures followed by Tukey's multiple-comparison test. There was a significant effect of alcohol withdrawal on the number of spindles (Figure 5A; Withdrawal state: $F_{(2,36)} = 3.53$, $p < 0.05$; Sex: $F_{(1,18)} = 0.42$, $p = 0.52$; Withdrawal state x sex: $F_{(2,36)} = 0.30$, $p = 0.73$). *Post-hoc* analyses revealed a significant increase in the number of spindles during protracted abstinence compared to acute withdrawal ($p < 0.05$). The number of spindles was further analyzed for each hour within the 12-hour period. There was no effect of alcohol withdrawal state on the number of spindles for each hour (Figure 5B; Withdrawal state: $F_{(2,38)} = 2.55$, $p = 0.09$; Time: $F_{(11,209)} = 1.43$, $p = 0.16$; Withdrawal state x time: $F_{(22,418)} = 1.29$, $p = 0.17$).

For spindle amplitude, there was a significant main effect of sex for spindle amplitude (Figure 5C; Withdrawal state: $F_{(2,36)} = 2.20$, $p = 0.12$; Sex: $F_{(1,18)} = 4.47$, $p < 0.05$; Withdrawal state x sex: $F_{(2,36)} = 1.19$, $p = 0.32$). This suggests that females had overall higher amplitude over the 12h period than males. Spindle amplitude was further analyzed for each hour within the 12 h period. There was a significant interaction of alcohol withdrawal state and time (Figure 5D; Withdrawal state: $F_{(2,38)} = 0.37$, $p = 0.69$; Time: $F_{(11,209)} = 2.7$, $p < 0.01$; Withdrawal state x time: $F_{(22,415)} = 2.62$, $p < 0.01$). *Post-hoc* analyses revealed a significant decrease in spindle amplitude during acute withdrawal compared to baseline at ZT 1 h only ($p < 0.01$).

1.6. REM and NREM sleep power spectral analysis

Spectral power was examined for REM and NREM sleep over the 12-hour recording using the MATLAB plugin EEGLAB (Delorme & Makeig, 2004; **Supplementary Figure 6**). The EEGLAB function spectopo uses the pwelch function; power spectrum density (PSD).

$$PSD = 10*\log10(\mu V/Hz)$$

The pwelch function used applied a hamming window with 0 overlap to calculate with a frequency resolution of 1 Hz. Relative power spectra plots were computed by taking the total absolute power spectra for each 1 Hz frequency and dividing absolute power for each frequency band by the overall total absolute power (sampling rate of 500/2). Relative spectral for 5 frequency bands (delta, 1-4 Hz; theta, 4-8 Hz; alpha, 8-13 Hz; sigma, 10-15 Hz; and beta, 16-30 Hz) was calculated as sum of the absolute power of a specific frequency band/the sum absolute power from 0.5 to 250 Hz.

The EEG power spectra for REM (Figure 6A) and NREM (Figure 6C) sleep epochs over the course of the light cycle (ZT 0-12h) for females (n = 12) and males (n = 8) were determined during baseline, acute withdrawal, and protracted abstinence. Relative spectral power was calculated for the 5 frequency bands was collapsed across alcohol withdrawal state. To examine potential sex differences in spectral power, a repeated measures two-way ANOVA with each band within a specific sleep state (REM or NREM) as the within-subjects factor and sex as a between-subjects factor followed by a Tukey's multiple-comparison test. There were no significant differences in REM power spectra between females and males (Figure 6A; REM, Sex: $F_{(1,58)} = 2.52$, p = 0.11; frequency band: $F_{(4,232)} = 57.41$, p < 0.01; Sex x frequency band: $F_{(4,232)} = 0.70$, p = 0.60), therefore subsequent analyses examined effects of alcohol withdrawal state on frequency bands. Power spectra was collapsed across sex and analyzed using a repeated measures two-way ANOVA with alcohol withdrawal state (baseline, acute withdrawal, and protracted abstinence) as the within-subjects factor and frequency band as a between-subjects factor. There was no effect of alcohol withdrawal state on REM power spectra in any of the 5

frequency bands (Figure 6B; Withdrawal state: $F_{(2,38)} = 0.39$, $p = 0.67$; Frequency band: $F_{(4,76)} = 52.84$, $p < 0.01$; Withdrawal state x frequency band: $F_{(8,152)} = 1.70$, $p = 0.10$).

For NREM power spectra, there were no significant differences between females and males (Figure 6C; Sex: $F_{(1,58)} = 0.62$, $p = 0.43$; Frequency band: $F_{(4,232)} = 477$, $p < 0.01$; Sex x frequency band: $F_{(4,232)} = 1.48$, $p = 0.21$), therefore subsequent analyses examined effects of alcohol withdrawal state on frequency bands. There was no effect of alcohol withdrawal state on NREM power spectra (Figure 6D; Withdrawal state: $F_{(2,38)} = 2.18$, $p = 0.12$; Frequency band: $F_{(4,76)} = 384.5$, $p < 0.01$; Withdrawal state x frequency band: $F_{(8,152)} = 1.79$, $p = 0.08$).

2. Supplementary Figures

2.1. Supplementary Figure 1.

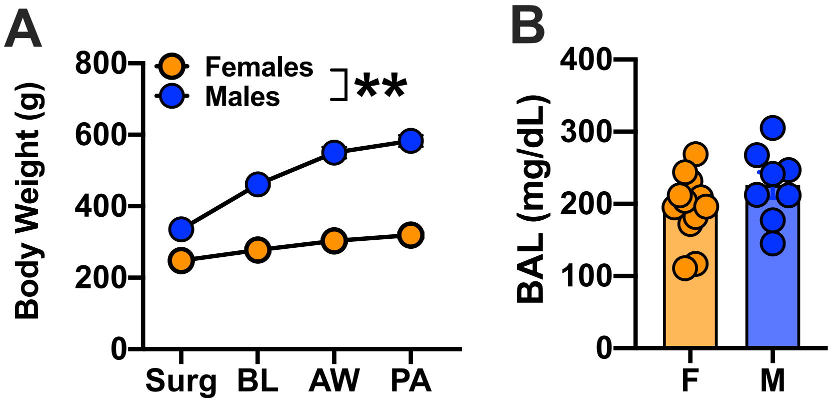

Supplementary Figure 1. (**A**) Changes in female (orange circles) and male (blue circles) bodyweights (g) prior to telemetry surgery (Surg), during baseline (BL), acute withdrawal from alcohol (AW), and following 4 weeks of protracted abstinence (PA). (**B**) Blood alcohol level (BAL; mg/dL) for females (F; orange bar) and males (M; blue bar) following four weeks of chronic intermittent ethanol vapor (CIEV) exposure. Despite a significant difference in female and male bodyweights, there was no significant difference between female and male BALs following four weeks of CIEV. $**p < 0.01$

2.2. Supplementary Figure 2.

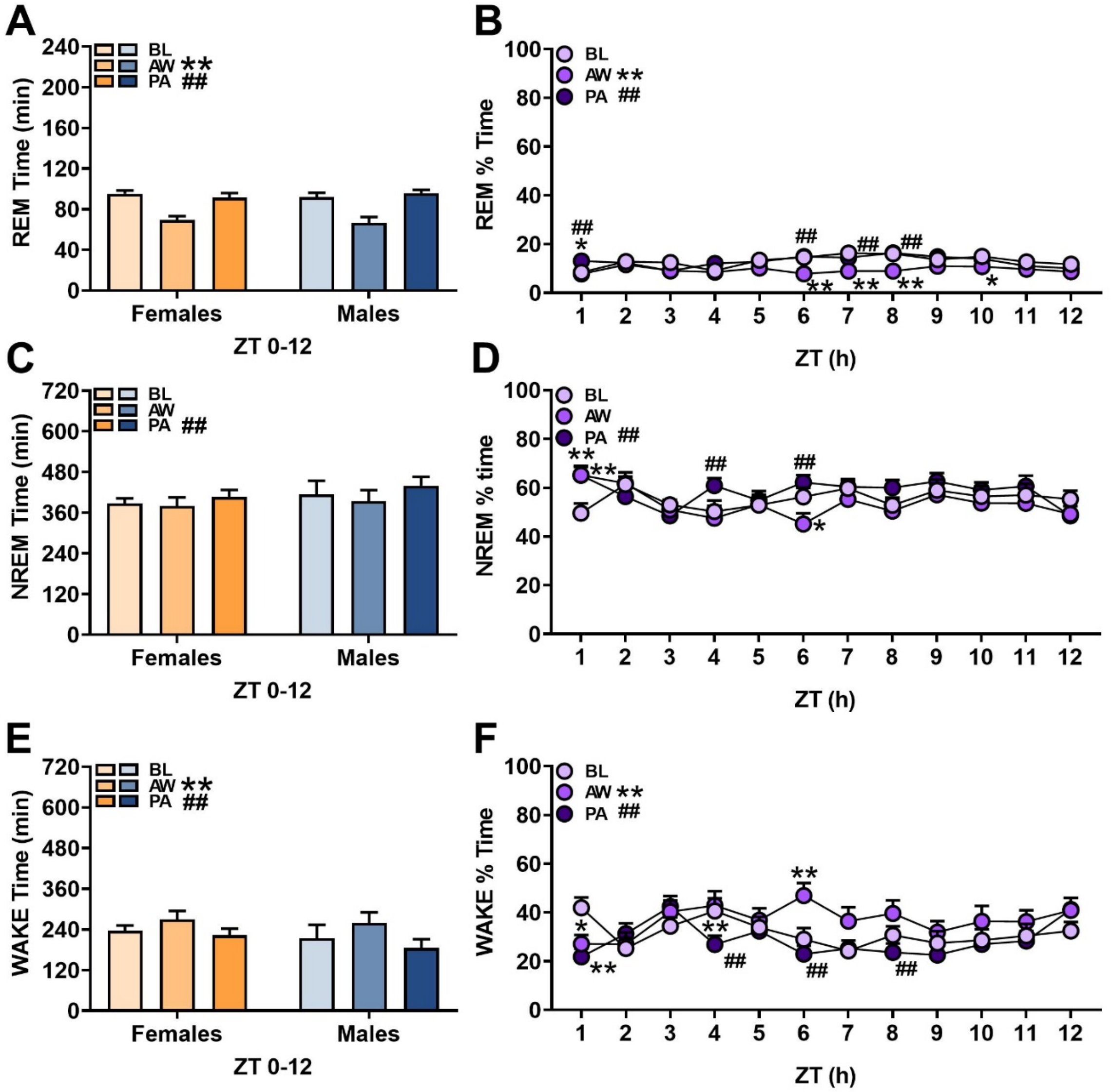

Supplementary Figure 2. (**A**: REM; **C**: NREM; **E** WAKE) Effects of ethanol withdrawal state (baseline [BL], acute withdrawal [AW], and 4 week protracted abstinence [PA]) on mean total time in minutes (+ SEM) spent in each sleep state) over the whole 12 h in females (orange bars), and males, (blue bars). (**B**: REM; **D**: NREM; **F** WAKE) Percent of time (+ SEM) spent in each sleep state was calculated for each hour (ZT 1-12) for females and males combined (BL: light purple circles; AW medium purple circles; PA: dark purple circles). *$p < 0.05$ versus BL; **$p < 0.01$ versus BL; $^{\#}p < 0.05$ versus AW; $^{\#\#}p < 0.01$ versus AW.

2.3. Supplementary Figure 3.

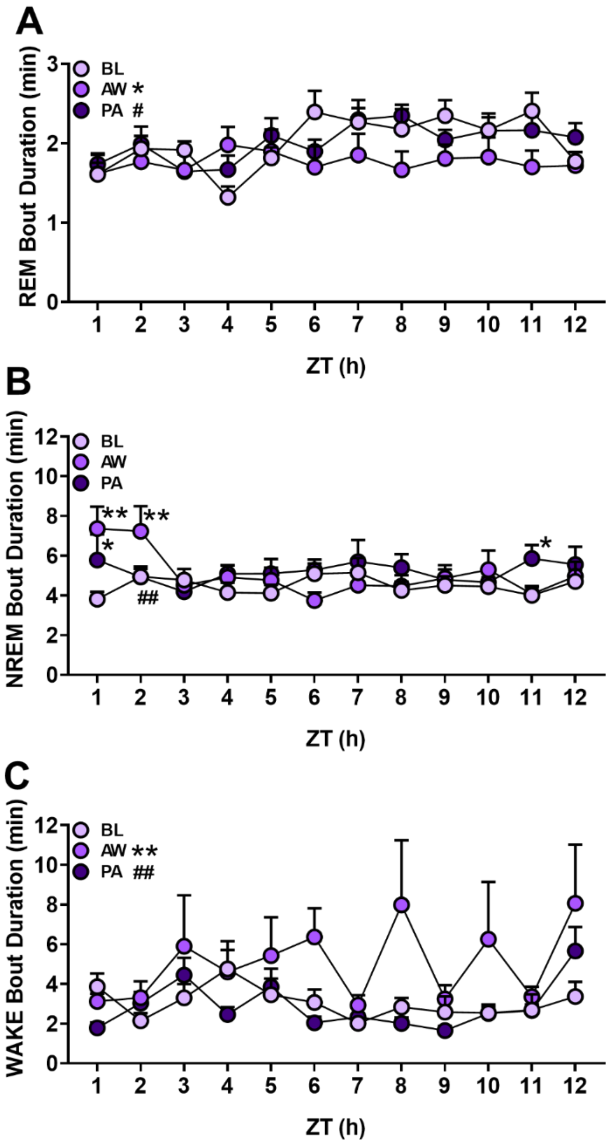

Supplementary Figure 3. (A, REM; **B**, NREM; **C**, WAKE) Effects of alcohol withdrawal state (baseline [BL]: light purple circles; acute withdrawal [AW]: medium purple circles; and protracted abstinence [PA]: dark purple circles) on bout duration (+SEM) for each hour (ZT 1-12) for females and males combined. $*p < 0.05$, $**p < 0.01$ *versus* BL; $^{\#}p < 0.05$, $^{\#\#}p < 0.01$ *versus* AW.

2.4. Supplementary Figure 4.

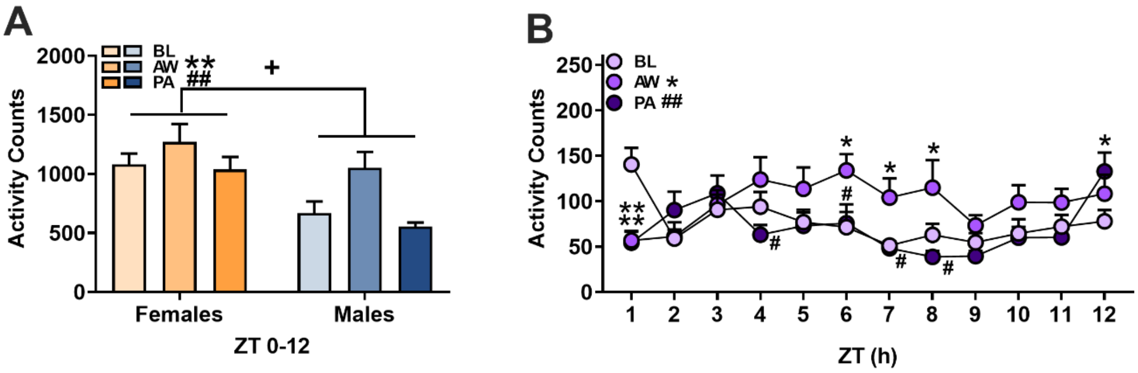

Supplemental Figure 4. (A) Effects of alcohol withdrawal state (baseline [BL], acute withdrawal [AW], and 4 week protracted abstinence [PA]) on activity counts (+ SEM) spent in each sleep state) over the whole 12 h in females (orange bars), and males, (blue bars). **(B)** Effects of alcohol withdrawal state on activity counts (+SEM) for each hour (ZT 1-12) for females and males combined (BL: light purple circles; AW medium purple circles; PA: dark purple circles). *$p < 0.05$, **$p < 0.01$ *versus* BL; $^{\#}p < 0.05$, $^{\#\#}p < 0.01$ *versus* AW.

2.5 Supplementary Figure 5.

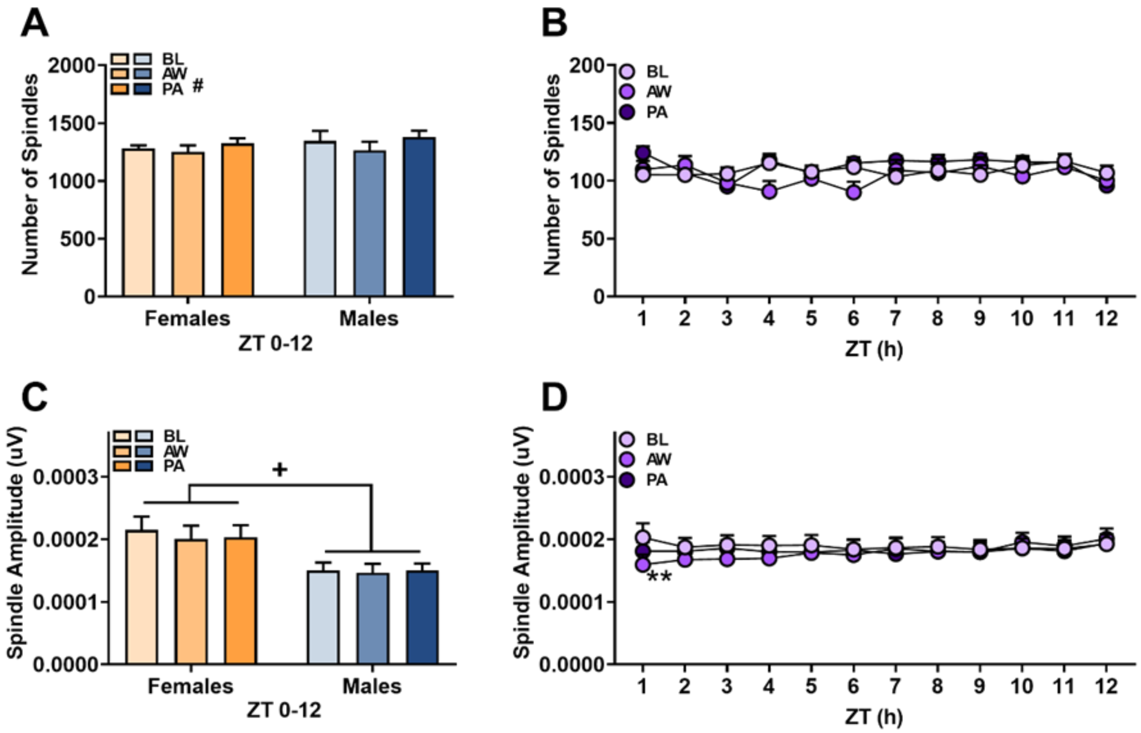

Supplemental Figure 5. (A and **B**) Effects of ethanol withdrawal state (baseline [BL], acute withdrawal [AW], and 4 week protracted abstinence [PA]) on the number of spindles (+SEM) over the 12 h period (**A**) in females (orange bars) and males (blue bars) and in each hour (**B**) for females and males combined (BL: light purple circles; AW medium purple circles; PA: dark purple circles). **(C** and **D**) Effects of ethanol withdrawal state on mean spindle amplitude in sec (+ SEM) over the 12 h period (**C**) in females (orange bars) and males (blue bars) and in each hour (**D**) for females and males combined (BL: light purple circles; AW medium purple circles; PA: dark purple circles). $^{+}p < 0.05$ versus males; $^{*}p < 0.01$ versus BL; $^{\#}p < 0.05$, $^{\#\#}p < 0.01$ versus AW.

2.6 Supplementary Figure 6.

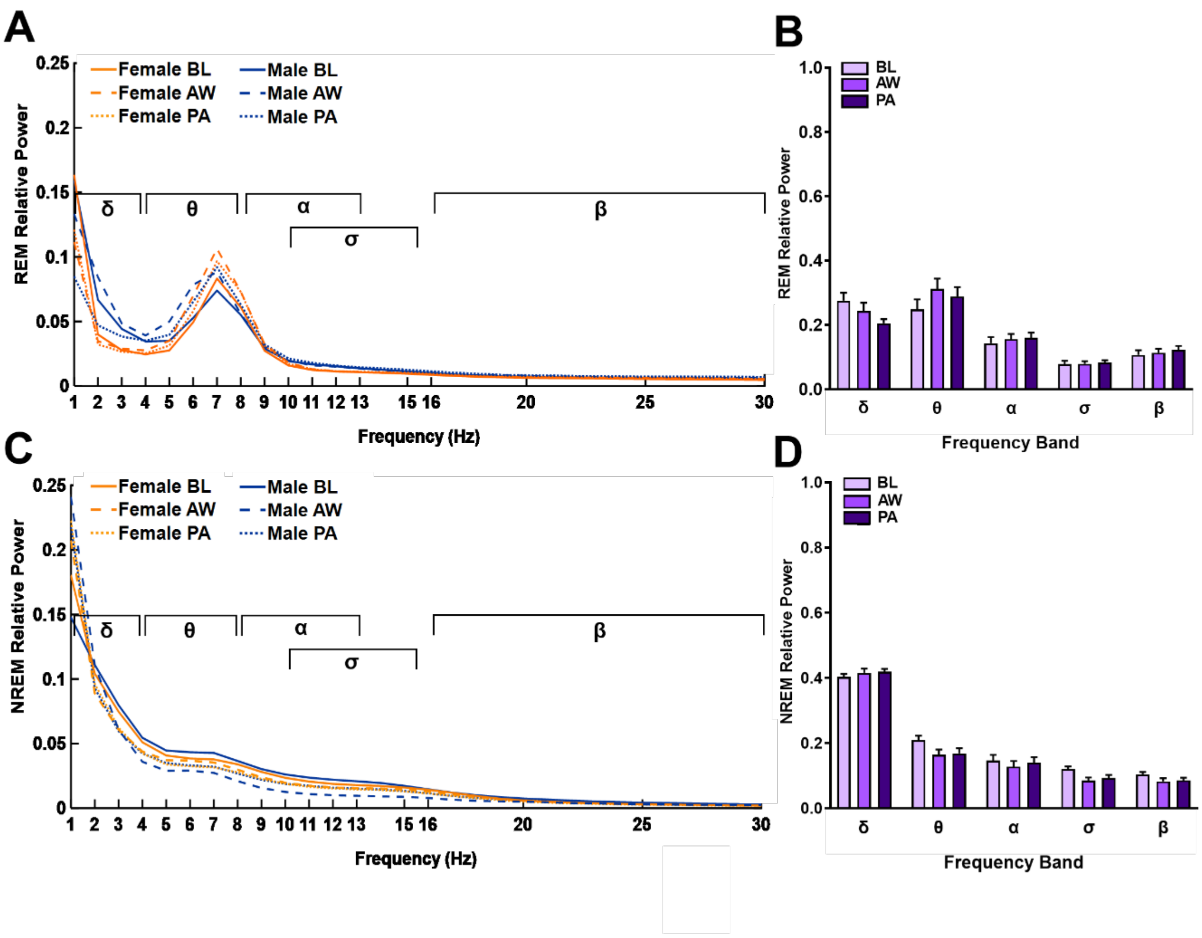

Supplementary Figure 6. (**A-B**, REM; **C-D**, NREM) Effect of alcohol withdrawal state (baseline [BL], acute withdrawal [AW], and protracted abstinence [PA]) relative EEG spectral power for in females (orange lines), males (blue lines), and combined (purple bars) for delta (δ), theta (θ), alpha (α), sigma (σ) and beta (β) frequency bands.
